# Greenhouse Gas Emission Transfer of Inter-Provincial Electricity Trade in China

**DOI:** 10.3390/ijerph17228375

**Published:** 2020-11-12

**Authors:** Wenbo Li, Ruyin Long, Linling Zhang, Zhengxia He, Feiyu Chen, Hong Chen

**Affiliations:** 1 Business School, Jiangsu Normal University, Xuzhou 221116, China; hezhengxia1979@jsnu.edu.cn; 2 School of Economics and Management, China University of Mining and Technology, Xuzhou 221116, China; chenfeiyu@cumt.edu.cn (F.C.); hongchenxz@cumt.edu.cn (H.C.); 3 School of Public Finance and Taxation, Nanjing University of Finance & Economics, Nanjing 210023, China; zll3474@nufe.edu.cn

**Keywords:** greenhouse gas, emission transfer, inter-provincial electricity trade, Quasi-Input-Output model

## Abstract

Inter-regional electricity trade is an important way to mitigate the imbalance between regional electricity generation and consumption. With the increasing amount of inter-regional electricity trade in China, the emission transfer problem is more severe. By using Quasi-Input-Output model, which can consider the ripple effect of electricity trade network, this study analyzed embodied greenhouse gas emissions of electricity trade among 30 provinces in China. Results indicated that, in 2017, the national transfer volumes of CO_2_, CH_4_, and N_2_O embodied in inter-provincial electricity trade were 603.25 Mt, 6803.81 t, and 9899.25 t, respectively. Emissions are mainly transferred from the eastern to the western regions, especially to those with high proportion of electricity generated from fossil fuels. The amount of emission transfer is not consistent with that of purchased electricity, since some regions are rich in clean energy. Although direct emission transfer plays the dominant role for most province, indirect emission transfer should also be noticed. Provinces with larger indirect emission transfer generally purchase electricity from provinces with a lot of electricity inflows. The findings could help policy makers coordinate regional energy utilization strategies and issue more effective emission reduction policies in the electricity industry.

## 1. Introduction

Electricity is the main power in promoting industrialization, urbanization and informatization of the world, and it is expected to continue playing an important role in social development for a long time [1]. According to the BP Statistical Review of World Energy, global electricity consumption in 2019 was 25.8 trillion kilowatt-hours (kWh) which accounts for approximately 20% of final energy consumption. However, electricity generation and consumption are inconsistent in regions due to different economic development levels, technological conditions and resource endowments [2]. To mitigate this spatial mismatch, electricity trade has become a valid method [3]. Large-scale electricity trade can optimize the allocation of energy resources, increase the utilization of electricity generation equipment, reduce the cost of electricity supply and ultimately improve the welfare of whole society [4]. International electricity trade developed rapidly in recent years. According to the International Energy Agency (IEA) statistics, there were more than 100 countries import electricity in the world, and the total amount of imported electricity was 725.92 terawatt-hours (TWh) in 2017 with an average annual growth rate of about 3% over the decade. In the future, the scale of international electricity trade will continue to expand and its share in electricity consumption and international energy trade will increase further.

Fossil fuels are the largest source of electricity, and by the end of 2018 coal, natural gas and oil contribute 64% of global electricity generation (about 17,049 TWh). Measures have been implemented to reduce the predominant role of fossil fuels, but it is difficult to change since the share of them in electricity generation just declines from 64.5% in 2000 to 64% in 2018. Even if the solar, wind, nuclear and other clean energy develop rapidly, fossil fuels can still be the mainstream of electricity supply for quite a long time [5]. Fossil fuels generation can bring serious emission problems. In 2018, CO_2_ emissions caused by coal, natural gas and oil were 9357, 2656 and 641 million tons (MT), respectively. The electricity grid is an open system, and in essence the electricity trade is similar to inter-regional product trade since they both contain embodied emissions [6]. For electricity imported regions, emissions of the imported electricity are stayed in electricity generation regions, and this can be interpreted as electricity imported regions transfer emissions to external regions. For electricity export regions, emissions of the exported electricity are undertaken by themselves. The essence of this problem is that emissions which should be undertaken by electricity consumption regions are transferred to generation regions [7]. Simply speaking, this is one kind of embodied pollution transfer problem. Pollution transfer is one of the most important environmental issues in the world today, and it refers to the phenomenon that a country or region undertakes the environmental loss and harm of others due to the geographical separation of product production and consumption caused by inter-regional trade. It is expected that emission transfer will be particularly prominent within the normalization of inter-regional electricity trade.

China is the world’s largest electricity producer and its electricity generation reached 7111.8 TWh in 2018, an increase of 7.7% over 2017, accounting for 26.7% of the world’s total electricity generation. Although the national electricity generation capacity is quite large, the electricity trade is still exited due to uneven geographical distribution of natural resources and regional economic disparity. Eastern coastal regions, which are of higher economic development level, account for over 60% of national electricity demand, but most provinces (e.g., Beijing, Tianjin, Jiangsu, and Zhejiang) are short of nature resources, thereby resulting in insufficient electricity generation capacity. By contrast, the central and western regions are of excess electricity generation capacity because of rich energy resources and low production cost. In order to mitigate the problem of electricity shortage in eastern coastal regions, large scaled thermal power stations have been constructed [8]. This measure rapidly decreases the environmental carrying capacity and further aggravates air pollution problems of these regions. For example, frequent fog and haze weather is a direct reflection of this. In 2013, an average of 30 days fog and haze weather affected more than 100 large and medium-sized cities, which seriously threaten public physical and mental health. In recent years, inter-regional electricity trade has become a normalization trend in China, and the transmission volume has maintained a steady growth [9]. For example, the exported electricity of Inner Mongolia was 1806 million kWh in 2017, accounting for 41% of local electricity generation; conversely, Guangdong imported a total of about 2227 million kWh of electricity which could satisfy 37% of local demand, and the imported electricity of Beijing and Shanghai even contributed to over half of local demand. From the report of China Electricity Council, inter-provincial electricity trade in China increased from 81.6 billion kWh in 2006 to 480.7 billion kWh in 2018, and its percentage in national electricity consumption increase from 2.85% to 7.03% accordingly [10]. So, it is expected that such electricity trades inevitably aggravate emission transfer problems among regions.

For one region, emissions of imported electricity are left in generation regions. Since they are not really released in electricity consumption regions, we define them as embodied emissions. Presently, embodied emissions of electricity trade have gained great concern. Watcharejyothin and Shrestha (2009) adopted a MARKAL-based model, which is the most predominant one in kinds of energy systems models [11], to investigate the impact of electricity trade between Laos and Thailand on pollutant emissions, and results showed that power trade is beneficial to mitigate pollutant emissions [12]. Qu et al. (2017) selected Eurasian Continent grid network as a case and analyzed the impact of inter-regional electricity trade. Results showed that, without considering embodied emissions in electricity trades there are mistakes in estimating emissions of regional electricity grids [13]. Through developing an optimization model for electricity generation and transmission system, MacDonald et al. (2016) found that long distance electricity transmission can help electricity generation in concert with renewables, nuclear and natural gas, thereby substantially reducing emissions in U.S. [2]. Similar results were also found by Jayadev et al. (2020), and they believe long distance electricity transmission is of great importance to emission reduction in the U.S. [14]. Orfanos et al. (2019) investigated the environmental performance of the Greek electricity sector, and results showed that the impact of the interconnected electricity trade system is relatively small, as compared to electricity generation [15]. There are also studies focused on embodied emissions of large-scale electricity trade in China. Su et al. (2017) found that inter-provincial electricity trade account for 14% of emissions of national electricity industry in 2012, and their study also showed that the emissions was transferred from the east to west regions [16]. Li et al. (2018) analyzed the pollution transfer effect of Yangtze River Delta region in 2015, and results indicated that 20.50 kt of SO_2_, 22.40 kt of NO_x_, 4.30 kt of dust, and 39.23 Mt of CO_2_ were transferred to generation-side regions (e.g., Shanxi, Sichuan and Hubei) by the electricity trade network [17]. Yi et al. (2020) further indicated that the embodied emissions of electricity trade are mainly transferred from the developed regions to resource-rich regions [18]. In other words, resource-rich regions undertake extra emissions for the electricity demand of other regions. Except for greenhouse gas (GHG) emissions and air pollution, there are similar studies focusing on other environmental problems. For examples, Zhang et al. (2017) analyzed virtual scarce water embodied in inter-provincial electricity trade in China [19]. Similarly, Wang et al. (2019) evaluated water scarcity risk caused by inter-regional electricity trade in China [20].

It can be seen that embodied emissions of inter-regional electricity trade have become a hot topic, so accurately accounting for the amount of such emissions is quite necessary. Based on electricity inflows and outflows of each province, the main objective of this study is to analyze the embodied emissions of inter-regional electricity trade in China. Exiting studies mainly concern embodied emissions caused by purchased electricity directly, but there are lack of considering parts of purchased electricity could be originated from other provinces. Therefore, the major contribution of this study is to provide an overview for embodied emissions of purchased electricity by considering the ripple effect of electricity trade network. Although the network approach has been considered by Qu et al. (2017) [7], direct and indirect components of transferred emissions are not clearly divided, which will be detailed explained in this study. The results of this study could provide empirical basis for the division of regional responsibilities in emission control, benefit the formulation of ecological compensation standard, and give implications for facilitating the comprehensive management of regional pollutions. This paper also provides an example for other countries or regions to account for embodied emissions of inter-regional electricity trade links the electricity trade network.

## 2. Methods and Data Collection

### 2.1. Methods

This study intends to adopt the following steps to achieve the research goal. First, based on electricity inflows and outflows of each province, we intend to establish an inter-provincial electricity trade network. Second, emission factors of provincial electricity consumption are then calculated by considering emissions of imported electricity and the electricity trade network. Finally, emissions transferred by inter-provincial electricity trade can be obtained by the Quasi-Input-Output (QIO) model, which is a general method that used to evaluate embodied emissions from generation to consumption through all transfer pathways in the whole network.

For most provinces, they are both electricity producers and consumers. However, the generated electricity of each province is not limited to local consumption, and a part of them is transferred through chains of grids and finally arrive at other provinces. For one province, the electricity outflows are the electricity inflows of other provinces, and vice versa. Such transmission relationships among regions can be expressed as the *n* by *n* matrix *T* which depends on the electricity flow relationships among provinces, and is shown below.

(1) $$T=\left[\begin{array}{cccc} 0 & T_{12} & \cdots & T_{1n}\\ T_{21} & \ddots & \ddots & T_{2n}\\ \vdots & \ddots & \ddots & \vdots \\ T_{n1} & \cdots & T_{n\left(n-1\right)} & 0 \end{array}\right]$$

where *T_nm_* represents the electricity transferred from region *n* to region *m*.

From the perspective of electricity generation, total electricity flow of one province is equal to the sum of local electricity generation and electricity inflows of other provinces. From the perspective of electricity consumption, the total electricity flow is local electricity consumption plus electricity outflows of this province. Thus, the total electricity flow can be taken as a balance point to establish relationships of provincial electricity inflows and outflows, as is shown in the following.

(2) $$x_{n}=p_{n}+\overset{q}{\underset{m=1}{\sum }}T_{mn}=c_{n}+\overset{q}{\underset{m=1}{\sum }}T_{nm}$$

where *x_n_* represents the total electricity flow of *n* province, *p_n_* and *c_n_* represent the electricity generated and consumed by *n* province, respectively.

Due to different power structure, emission factors of imported electricity and generated electricity are not same. Emission factors of generated electricity cannot be directly used to obtain transferred emissions of inter-provincial electricity trade since imported and exported electricity are commonly exited in most provinces. Thus, it is necessary to calculate emission factors for electricity consumption through Equation (3). It can be seen that the numerator is emissions of generated electricity plus emissions of imported electricity from other provinces, and the denominator is the total electricity flow of one provincial grid.

(3) $$f_{ni}=\frac{e_{ni}^{p}+\sum_{m=1\;m\neq n}^{q}T_{mn}e_{mi}^{x}}{x_{n}}$$

where *f_ni_* denotes the factor of emission *i* of grid *n*, and *e^p^_ni_* and *e^x^_mi_* are the amount of emission *i* of generated electricity for grid *n* and total electricity flow for grid *m*, respectively.

For one province, emissions of imported electricity are left in electricity generation provinces. Thus, emissions transferred by inter-provincial electricity trade can be defined as emissions of imported electricity. Since there are parts of power loss in the transmission process, emissions of this part should also be considered. Based on the electricity trade network and emission factors of electricity consumption, transferred emission of provincial grids can be calculated through the QIO model, and the amount of transferred emissions can be expressed as Equation (4). The detailed steps can be found in the study of Qu et al. (2017) [13].

(4) $$e_{ni}^{t}=\overset{q}{\underset{m=1\;m\neq n}{\sum }}T_{mn}\times f_{mi}$$

where *e^t^_ni_* denotes the amount of emission *i* caused by imported electricity of province *n*, and *q* is the number of provincial power grids. 

### 2.2. Data Collection

**Figure 1:**
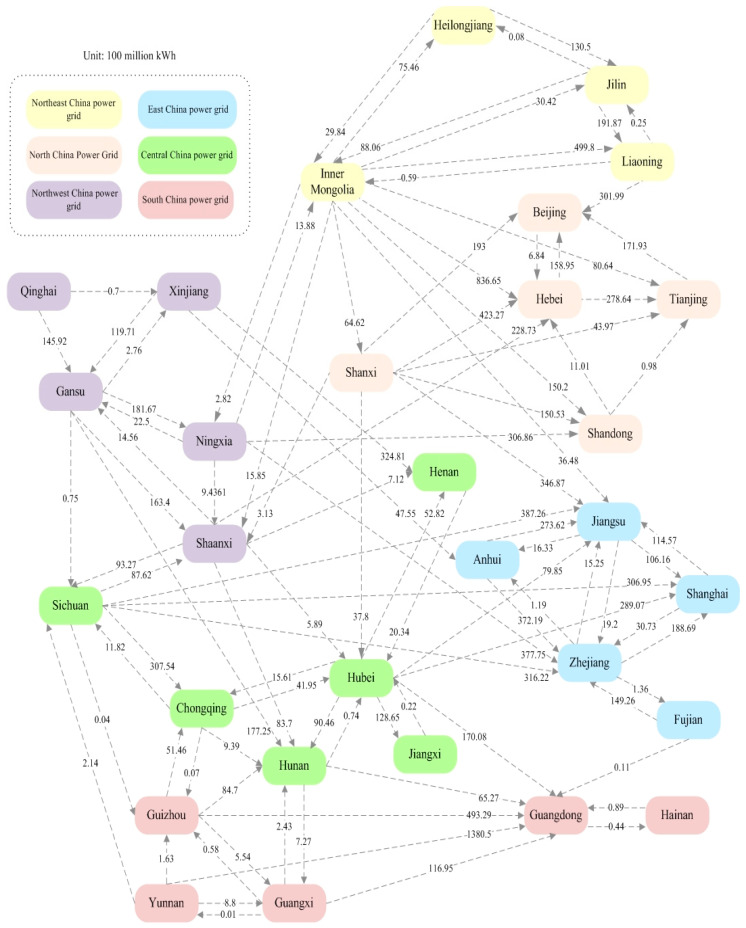
Inter-provincial electricity trade in China, 2017.

In this study, 30 provinces are included to calculate embedded emissions of inter-provincial electricity trade. Tibet, Hong Kong, Macao and Taiwan are excluded because of incomplete public data. Since fossil fuels are the main power source for most regions in China, kinds of GHG emissions are produced in the electricity generation process. Based on the global climate report of World Meteorological Organization, we selected CO_2_, methane (CH_4_) and nitrous oxide (N_2_O) to reflect GHG emissions. Emissions of one electricity grip basically equal to that of all kinds of fossil fuels consumed in the electricity generation process. Since emissions of electricity generation for the wind, hydropower, and solar power are relatively lower, they are not considered in this study. The data of electricity generation, station operation power consumption rate and energy structure for electricity generation are obtained from China Electric Power Yearbook [21]. Imported and exported electricity of each province is gained from Compilation of Statistical Data of Power Industry [10] and Annual Development Report of China’s Electric Power Industry [22]. China Energy Statistical Yearbook [23] provides the fuel consumption in electricity generation and average heating value. Based on the collected data, the amount of electricity trade among provinces in 2017 are presented in Figure 1.

## 3. Results

**Figure 2:**
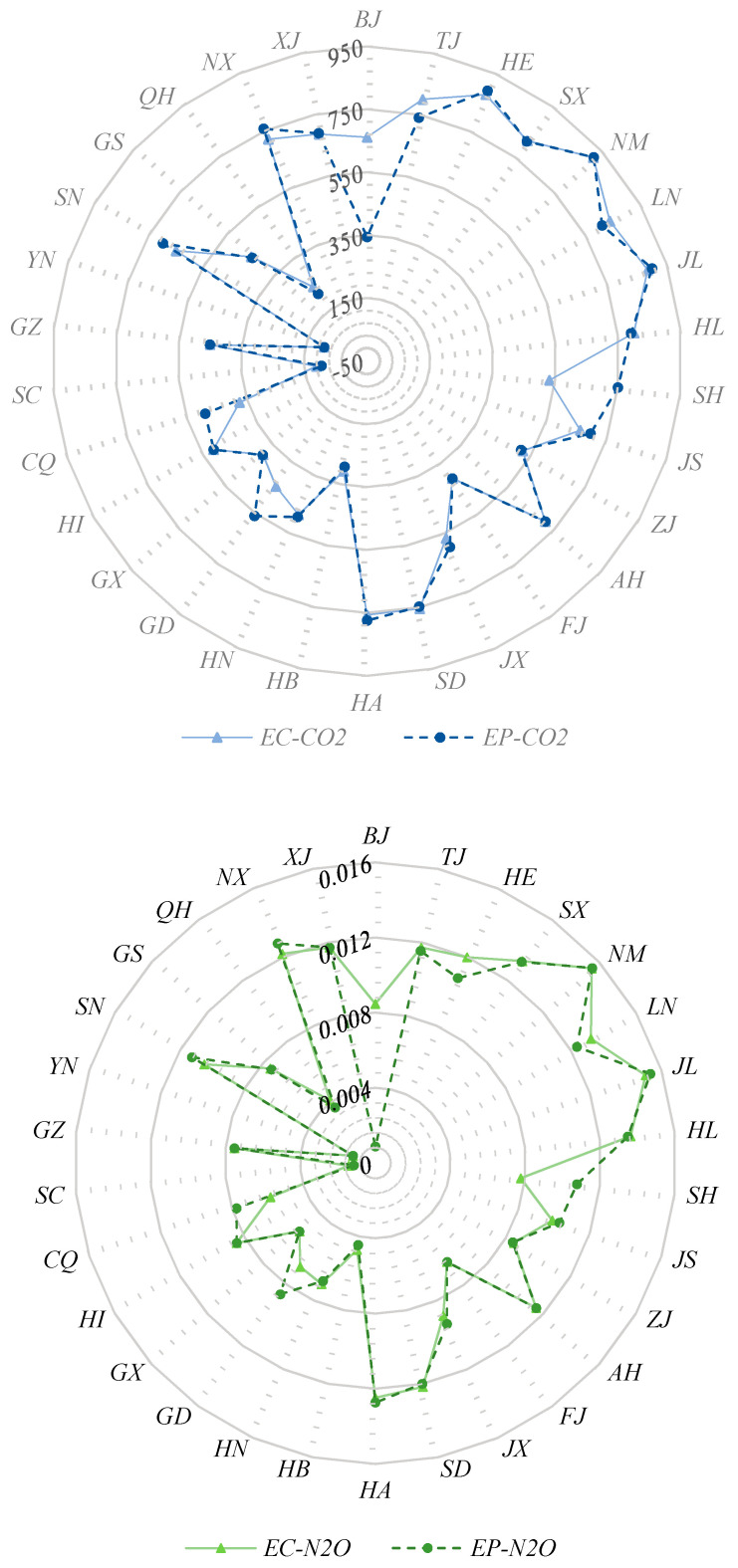

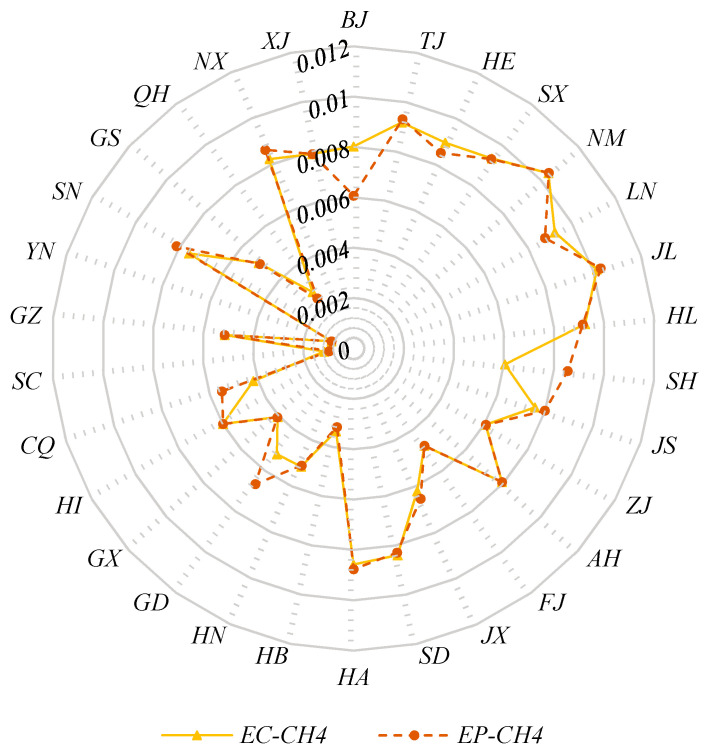
Emission factors of provincial electricity grids (Unit: g/kWh). Note: EC and EP denote emission factors of electricity consumption and generation, respectively.

Emission factor of provincial electricity grid is a necessary index to calculate transferred emissions, and it is defined as the proportion of emission caused by electricity generation or consumption. Emission factor of electricity generation is easier to obtain, and it is widely used in existing studies. When there is low imported electricity, emission factors of electricity generation and consumption are basically same. With the increasing amount of inter-regional electricity trade, emissions of imported electricity cannot be ignored since transferred emissions are mainly caused by the consumption of imported electricity. Therefore, we calculated emission factors of electricity consumption after considering the imported electricity, and the results can be seen in Figure 2. We can see that emission factors of electricity generation and consumption for most provinces differ a lot. For example, emission factors of electricity consumption are higher than that of electricity generation in Beijing for the following reasons. In Beijing, power plants are of high generation efficiency and emission reduction ability, and there is a low amount of coal in the power structure of electricity generation. So, emission factors of electricity generation in Beijing are lower than surrounding provinces. After considering the imported electricity, emission factors of electricity consumption increase. The main reason is that the biggest electricity importers of Beijing are Mongolia and Shanxi, and coal is dominated in the local fuel mix of both provinces. Conversely, emission factors of electricity consumption would be decreased if the imported electricity mainly comes from regions where the power structure is dominated by non-fossil energy. Guangdong is an example because its imported electricity mainly comes from Yunnan where the proportion of hydro power is 84.58%. Similar situation is existed in Jiangsu, Zhejiang, and Shanghai since they import electricity from Sichuan which is also rich in water resources. Provinces in the northeast and north China generally have higher emission factors, while provinces with lower emission factors are mainly located in the central and western China. Emission factor of CO_2_ is much higher than that of N_2_O and CH_4_ for all provinces. Provinces which have higher emission factor of CO_2_ (above 750 g/kWh) are Tianjin, Hebei, Shanxi, Inner Mongolia, Liaoning, Jilin, Heilongjiang, Shandong and Henan. Emission factors of N_2_O for Beijing, Tianjin, Hebei, Inner Mongolia, Liaoning and Jilin are above 1 g/kWh, which are higher than those of other provinces. Tianjin, Shanxi, Liaoning, Jilin and Heilongjiang have higher emission factors of CH_4_ (above 0.009 g/kWh). Higher emission factors mean more emissions embodied in exported electricity, which can result in higher emissions of electricity imported in provinces.

Based on the QIO model, transferred emissions caused by inter-provincial electricity trade are obtained. To sum up, national volume of CO_2_, CH_4_, and N_2_O embedded in inter-provincial electricity trade is 603.25 Mt, 6803.81 t, and 9899.25 t, respectively. Main emission exported and imported provinces can be seen in Table 1 and Table 2. Emission exported provinces (e.g., Beijing, Zhejiang, Shanghai and Jiangsu) are mainly located in eastern China, while emission imported provinces (e.g., Inner Mongolia, Shanxi, Ningxia and Xinjiang) are mainly distributed in western China. That is to say, general emission transfer direction is from the eastern to the western China. Hebei is the largest emission exported province, followed by Beijing, Zhejiang and Jiangsu, and the total amount of their exported emissions is more than half of national volume. The electricity demand of these provinces increases largely in recent years, and their electricity supply capacity is insufficient due to low environmental carrying capacity. In order to mitigate this problem, the operation of West–East electricity transmission project plays an important role in meeting the growing electricity demand of these provinces. Inner Mongolia and Shanxi are the two biggest emission imported provinces and their combined percentage of imported emissions is over 40% of national level. Among all provinces, Inner Mongolia is the largest emission importer, and main reasons can be summarized as the following two aspects. First, Inner Mongolia is rich in nature resources, and in 2017 its electricity generation capacity is 442.4 billion kWh. Second, although there are rich hydro, solar and wind electricity generation capacity in Inner Mongolia, local electricity generation structure is dominated by fossil fuels which accounts for 84.45% of total generation capacity.

Through the QIO model, transferred emissions can be divided into two parts, namely direct and indirect transferred emissions. Direct transferred emissions are caused by the purchased electricity. If the purchased electricity contains the electricity that is purchased from other regions, this part produces indirect transferred emissions. In order to present the advantage of QIO model, direct transferred emissions are provided to compare with the total amount in Table 1 and Table 2. It can be seen that without considering the indirect part, transferred emissions are not accurate for most provinces. There are also exceptions, such as Hebei. The total and direct imported emissions of this province are same, which means there are no indirect transferred emissions. This is mainly because Hebei mainly exports electricity to Beijing and Tianjin, and both export few electricity to other provinces.

Main emission transfer pathways are provided in Table 3. Through north channels of West–East electricity transmission project, Inner Mongolia, Shanxi, Shaanxi and Ningxia are main destinations of emissions transferred from Beijing, Tianjin, Hebei and Shandong. Guangdong transfer a lot of emissions to Guizhou though south channels of West–East electricity transmission project. Other chief pathways are basically existed in one regional electricity grid. For example, lots of emissions are transferred from Jiangsu and Zhejiang to Anhui, and they all belong to East China Electricity Grid. Among all, the pathway from Hebei to Inner Mongolia is the most prominent, and the amounts of transferred CO_2_, CH_4_, and N_2_O are 67.85 mt, 770.46 t, and 1145.99 t, respectively. It is noted that main emission exported provinces basically need large amount of imported electricity. However, provinces (e.g., Yunnan, Sichuan and Gansu) which export a large amount of electricity are not all major emission importers. For example, the amount of electricity Guangdong purchased from Yunnan is 138.05 billion kWh, which is more than half of all imported electricity, but the embedded emissions only account for about 20% of transferred emissions. The main reason is that the electricity supply structure of these provinces is dominated by non-fossil energy (i.e., hydropower, wind and solar). To be specific, Non-fossil energy of Yunnan, Sichuan and Gansu account for 91.89%, 90.08% and 47.32% of their electricity generation capacity in 2017, which significantly reduce the emissions of exported electricity.

## 4. Discussion

The main aim of this study is to analyze GHG emission transfer of inter-provincial electricity trade in China. In order to realize this, emission factors of electricity consumption in 30 provinces are obtained. Compared with previous studies, which adopted earlier public data, such as Ma et al. (2014) [24], Li et al. (2018) [17] and Yi et al. (2020) [18], emission factors of most provinces decrease and the reasons can be summarized as follows. First, the government continues to promote the consumption of non-fossil energy which benefit emission reduction in power plants. The Annual Development Report of China’s Electric Power Industry shows that the proportion of non-fossil energy consumption in China has increased from 12.1% in 2015 to 13.8% in 2017, and it is expected to reach 15% in 2020 [10]. Second, the electricity industry is an important source of GHG emissions, so great efforts have been done to improve the efficiency of electricity generation, such as shutting down small thermal power stations, improving the utilization rate of pollutions, and adopting high-efficiency electricity generation technologies. It is noted that the standard coal consumption of China’s coal-fired power plants with 6000 kw or above generation capacity is 309 g/kWh, which is an advanced record in the world. Third, the government encouraged the construction of ultra-high voltage transmission lines. By the end of 2017, 12 ultra-high voltage transmission lines were constructed and put into operation in China, and there are still several under construction. 

**Figure 3:**
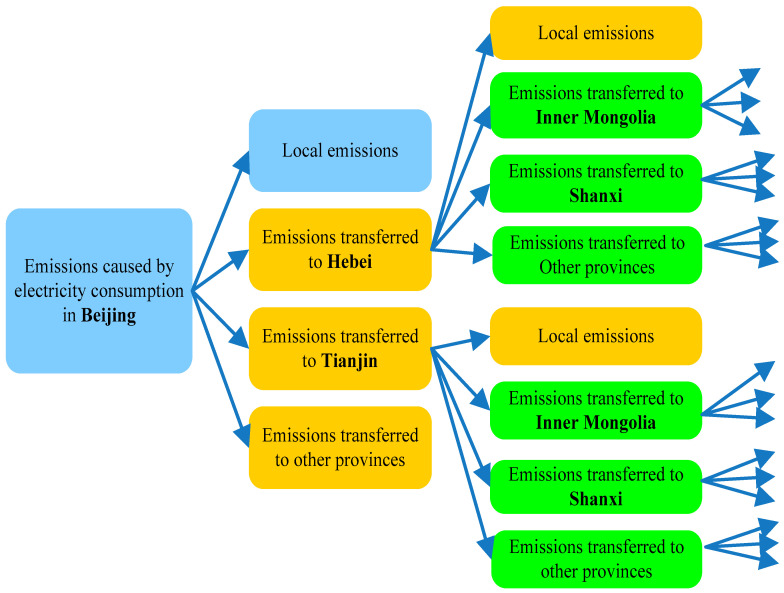
The structure of emissions caused by electricity consumption in Beijing.

After obtaining provincial emission factors of electricity consumption, transferred emissions are calculated through QIO model. Compared with previous studies, the ripple effect of national electricity trade network is taken into consideration in this model. Based on the results, it can be seen that although direct transferred emissions play an important role, the indirect part cannot be ignored. For example, indirect transferred emissions of Beijing account for 80% of the total amount. Figure 3 presents the structure of emissions caused by electricity consumption in Beijing. We can see that except for local emissions, emissions are directly transferred to electricity exporters (e.g., Hebei and Tianjin). Emissions of electricity consumption in Beijing can be indirectly transferred to Inner Mongolia and Shanxi Since they are electricity exporters of Hebei and Tianjin. That is to say, if the purchased electricity contains the electricity that comes from other grids, we cannot regard the emissions per unit of them as same. In order to get accurate results, overall electricity flows among provincial grids are needed before the establishment of QIO model. In China, transferred emissions undoubtedly have a great negative impact on the central and western regions with large electricity transmission capacity, but we should note that this impact largely depends on the energy scenario. In the short term, it is difficult for China to move toward an energy structure dominated by non-fossil fuels. Thus, under such a scenario, transferred emissions are consistent with the development of inter-regional electricity trade. On the contrary, if the leading role of fossil fuels in energy structure is replaced by non-fossil fuels, transferred emissions of inter-regional electricity trade will be reduced, which benefit both electricity importers and exporters.

The general situation of transferred emissions caused by inter-provincial electricity trade is similar with that of previous studies, and it is analyzed from the following aspects: political institution, economic development, population density, resource endowment and environmental cost. First, as long as all provinces in China are under the restriction of national environmental standards, provincial electricity trade can be realized through the market. Even if there are some problems, under China’s unified political institution they can be solved by administrative coordination [25]. This is the institutional basis for inter-provincial electricity trade. Second, electricity demand is often positively correlated with economic development and population density, which means developed provinces with dense population are generally of higher electricity demand [26]. In China, these provinces are mainly distributed in eastern coastal regions, where energy resources are insufficient and electricity generation capacity cannot meet local demand. Third, environmental costs vary greatly among regions. The environmental cost of electricity generation in eastern regions is relatively higher due to their developed economy, dense population and insufficient energy resources [27]. On the contrary, abundant energy resources, less population and undeveloped economy lead to lower environmental cost of electricity generation in western regions. According to the studies of Xu (2011), environmental loss of electricity generation in western regions is approximately twice as high as in eastern regions [28]. The western regions export a large amount of electricity to eastern regions, so that the corresponding emissions are transferred to the region with lower environmental cost, which is beneficial to the minimization of national environmental cost. Inter-regional electricity trade can not only meet the rapidly growing electricity demand of eastern regions, but also make full use of resource advantages and promote economic development of western regions [12]. All these factors determine that transferred emissions shows a trend from the east to the west. Apart from the above analysis, it is noted that the influence of transferred emissions on each province is different. We standardized the transferred emissions of major provinces, taking into account their area and population, and the results can be found in Table 4. In order to make the results easily comparable, we aggregated CO_2_, CH_4_ and N_2_O into GHG emissions by using the global warming potential. We can see that, after considering the area and population, transferred emissions of Beijing, Jiangsu, Tianjin, and Shanghai improve a lot, and this is influenced by their large population and small area. It can be interpreted that, as the environmental effect of transferred emissions for regions are different, regions with more population and small area benefit more due to their limited environment carrying capacity. The most obvious example is Shanghai, whose transferred emissions before and after standardization are 48.98 and 394.82 Mt, respectively. By contrast, transferred emissions of Hebei and Liaoning decrease, and this suggested that provinces with small population density benefit less due to transferred emissions caused by inter-regional electricity trade.

Based on above results, some implications are provided below. First, the government could improve the proportion of electricity generated by non-fossil energy (e.g., hydropower, wind and solar) in inter-regional electricity trade. The external cost of non-fossil energy is low, and they are mainly located in the southwest and northwest China. Through improving transmission capacity of electricity generated by non-fossil energy, the growing electricity demand of eastern regions (such as more electric vehicles are adopted [29]) can be satisfied and the emission transfer problems can also be alleviated. Second, the government could improve the efficiency of thermal power units and promote the widespread use of emission reduction devices. Increasing the scale of thermal power units can significantly improve the coal combustion efficiency and reduce the unit cost of applying advanced environmental protection technologies, thereby achieving low coal consumption and reducing the environmental impact on electricity exported regions. Third, the government could improve the transmission efficiency and reduce the power loss of transmission channels. At present, a large number of long-distance electricity transmission projects have been built, but they cannot be effectively utilized thereby resulting in low transmission efficiency. Therefore, more reasonable arrangements are needed to offset this defect. Finally, the government could establish regional ecological compensation mechanisms. The former three methods could reduce emissions of electricity generation but cannot make up environmental losses. The problem that who should be responsible for the embodied emissions is very important for policy making since it is relevant to decompose national emission reduction targets into regional responsibilities. At present, the principle “who benefits, who pays” is widely recognized, and it denotes that emission exported provinces should undertake the responsibility. Although the electricity price has been paid, this cost does not include environmental and health losses of electricity generation regions. Therefore, it is necessary to establish ecological compensation mechanisms to coordinate the environmental and health losses of different regions and alleviate their conflicts. Presently, feasible compensation mechanisms include direct economic compensation, environmental tax and emission trading. For example, direct economic compensation is more suitable for emission imported regions where electricity generation structure is dominated by fossil fuels. Such compensation can be used to purchase emission reduction equipment for thermal power units, which could directly produce environmental benefits.

## 5. Conclusions

The unbalanced spatial distribution of nature resources and economic development level in China leads to the gap of regional electricity generation and consumption, which causes inter-regional electricity trade as well as the corresponding GHG emission transfer problem. On the basis of collecting inter-provincial electricity flows, transferred emissions were evaluated by using the QIO model, which can trace indirect transferred emissions. The results showed that national transfer volume of CO_2_, CH_4_, and N_2_O embodied in inter-provincial electricity trade is 603.25 Mt, 6803.81 t, and 9899.25 t, respectively. Emissions are mainly transferred from the eastern developed regions to the western underdeveloped regions, especially to those with high proportion of electricity generated from fossil fuels (e.g., Shanxi, Inner Mongolia, Anhui, Ningxia). Provinces with a large amount of exported emissions are basically major electricity imported provinces (e.g., Beijing, Tianjin, Hebei, Liaoning, Shanghai, Jiangsu, Zhejiang, Shandong and Guangdong). However, some provinces (e.g., Yunnan, Sichuan and Gansu) with a large amount of exported electricity are not large emissions importers due to their cleaner electricity generation structure. This study provides evidence to prove that the ripple effect of electricity trade should be taken into consideration in analyzing emission transfer problems. If there are valuable data, more detailed results, such as emission transfer of inter-city electricity trade, can be analyzed. In addition, we just consider GHG emissions of electricity generation process in this study, and a further study is needed to analyze how the transferred emissions influence the results of life cycle analysis.

## Figures and Tables

**Table 1:** Major emission exported provinces.

No.	Province	CO_2_ (Unit: Mt)		CH_4_ (Unit: t)		N_2_O (Unit: t)	
		Total	Direct	Total	Direct	Total	Direct
1	Hebei	113.91	111	1299.91	1266.67	1894.61	1845.77
2	Beijing	68.25	54.35	755.28	597.02	1047	812.42
3	Zhejiang	63.2	60.06	711.92	676.01	1022.97	970.52
4	Jiangsu	62.75	61.29	705.91	689.03	995.79	971.60
5	Liaoning	55.17	53.06	628	603.64	934.25	898.32
6	Shandong	48.1	46.24	548.24	526.97	805.12	773.62
7	Guangdong	48.14	47.22	520.82	510.36	734.59	719.59
8	Tianjin	29.32	28.22	309.85	297.09	428.76	410.80
9	Henan	24.21	24.07	277.28	275.7	409.84	407.58
10	Shanghai	26.49	22.26	297.34	249.34	404.19	335.04

**Table 2:** Major emission imported provinces.

No.	Province	CO_2_ (Unit: Mt)		CH_4_ (Unit: t)		N_2_O (Unit: t)	
		Total	Direct	Total	Direct	Total	Direct
1	Inner Mongolia	159.52	148.29	1811.47	1683.91	2694.43	2504.68
2	Shanxi	95.21	93.79	1089.06	1072.85	1550.46	1527.38
3	Ningxia	48.83	46.94	556.67	535.12	824.44	792.51
4	Anhui	44.76	43.48	498.28	493.99	723.22	702.48
5	Xinjiang	33.77	29.84	387.97	342.78	576.24	509.12
6	Guizhou	28.66	24.87	327.17	283.86	478.34	415.02
7	Hebei	24.82	24.82	236.91	236.91	300.21	300.21
8	Hubei	23.28	23.11	253.06	251.27	353.54	351.03
9	Shaanxi	25.6	21.77	297.92	253.39	413.01	351.28
10	Jilin	20.89	17.34	239.08	198.42	356.32	295.72

**Table 3:** Major transfer pathways of CO_2_, CH_4_ and N_2_O.

No.	CO_2_ (Unit: Mt)		CH_4_ (Unit: t)		N_2_O (Unit: t)	
1	Hebei → Inner Mongolia	67.85	Hebei → Inner Mongolia	770.46	Hebei → Inner Mongolia	1145.99
2	Liaoning → Inner Mongolia	39.81	Liaoning → Inner Mongolia	452.12	Liaoning → Inner Mongolia	672.49
3	Hebei → Shanxi	30.22	Hebei → Shanxi	345.74	Hebei → Shanxi	492.22
4	Jiangsu → Shanxi	27.07	Jiangsu → Shanxi	309.57	Jiangsu → Shanxi	440.73
5	Zhejiang → Anhui	24.75	Zhejiang → Ningxia	276.22	Zhejiang → Ningxia	409.09
6	Zhejiang → Ningxia	24.23	Zhejiang → Anhui	275.57	Zhejiang → Anhui	399.97
7	Guangdong → Guizhou	22.43	Guangdong → Guizhou	256.07	Henan → Xinjiang	378.19
8	Henan → Xinjiang	22.17	Henan → Xinjiang	254.63	Guangdong → Guizhou	374.39
9	Shandong → Ningxia	20.57	Shandong → Ningxia	234.52	Shandong → Ningxia	347.34
10	Jiangsu → Anhui	18.72	Jiangsu → Anhui	208.43	Jiangsu → Anhui	302.52

**Table 4:** Transferred GHG emissions before and after standardization (Unit: Mt).

Provinces	Transferred GHG Emissions before Standardization	Transferred GHG Emissions after Standardization
Hebei	226.08	190.19
Beijing	123.18	334.22
Zhejiang	123.88	144.28
Jiangsu	124.65	204.85
Liaoning	108.80	68.41
Shandong	94.83	129.55
Guangdong	95.81	124.84
Tianjin	57.80	167.23
Henan	48.53	58.34
Shanghai	48.98	394.82
